# Micromechanical BEoL robustness evaluation methods enabling loading condition customization and acoustic emission damage monitoring^☆^

**DOI:** 10.1016/j.mex.2023.102028

**Published:** 2023-01-19

**Authors:** Jendrik Silomon, Dulguun Chimeg, André Clausner, Ehrenfried Zschech

**Affiliations:** a Technical University Dresden, Helmholtzstr. 10, 01069 Dresden, Germany; b Microelectronics and Nanoanalytic, Fraunhofer Institute for Ceramic Technologies and Systems IKTS, Maria-Reiche-Strasse 2, 01109, Dresden, Germany; c deepXscan GmbH, Zeppelinstr. 1, 01324 Dresden, Germany

**Keywords:** Sub-critical BEoL damage induction and analysis, Cu-pillar tensile stress testing, Individual Cu-pillar soldering, Flexible customization of micromechanical load induction to individual electrical microchip connectors for BEoL robustness evaluation

## Abstract

For micromechanical robustness evaluation methods, it is advantageous if the mechanical loading conditions applied can be controlled as precisely as possible. For microchips, this is required to determine the robustness under specific conditions, e.g. during assembly or characteristic application/usage scenarios. In this work, three different micromechanical BEoL (Back End of Line) robustness evaluation methods are presented which should enable a more precise and flexible mechanical load induction and damage identification. They have been subsequently developed. Three main aspects characterize the customization of the developed approaches:

• The design and testing of customized micro-tools to precisely apply mechanical load to individual Cu-pillars.
• The implementation of an AE (Acoustic Emission) monitoring approach to detect minor damages during mechanical loading. This strategy also enabled the development of sub-critical loading experiments for which AE signals served as a damage indicator and mechanical loading was aborted upon the detection of AE events.
• The development of a new measurement setup and approach to enable the solder attach of individual Cu-pillars to a mechanical testing system.

The applications of these approaches should enable the induction of customized mechanical loading conditions and the identification of failure modes and damage initiation locations.

## Graphical abstract

**Figure:**
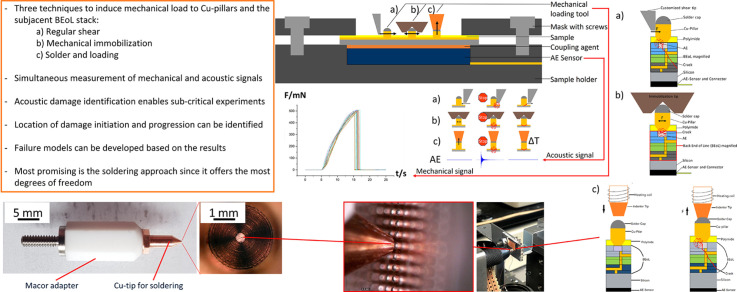

Specifications table

**Table:** 

Subject area:	
More specific subject area:	Semiconductor Robustness Testing
Name of your method:	Flexible customization of micromechanical load induction to individual electrical microchip connectors for BEoL robustness evaluation
Keywords:	*BEoL robustness* *Micromechanical testing* Acoustic emission * ndividual Cu pillar soldering
Original Method:	*n.A.*
Resource availability:	*n.A.*
Review question:	*- Is it possible to emulate the assembly process on single Cu-pillars?* *- Can the soldering approach be utilized to develop customizable BEoL robustness testing approaches?* *- Can Acoustic Emission be utilized to identify intermediate BEoL stack damage states?*

## Method details

The overall objective of the methods presented in this work is the determination of BEoL failure modes and the related conditions under which they occur by inducing mechanical load to adjacent electrical connectors. Different methods have been developed and successfully implemented in the industry to serve this purpose, such as the BABSI (Bump assisted BEoL stability indentation) test as well as shear tests [1], [2], [3]. Also, the JEDEC standard JESD22-B117 should serve this purpose [4], it is only applicable to solder balls, however. These methods work well for specific cases, but they don't offer many degrees of freedom regarding the individual adaption to specific samples or the precise control of the components of the stress fields in a BEoL stack. Due to these limitations, it was concluded that the methods could only emulate the mechanical condition during assembly or application, under which specific damage occur, to a certain extent. Also, it is difficult to determine the exact location of damage initiation and the propagation trajectory through the BEoL stack by deploying these methods. They rely mainly on the measurement of mechanical data which is not necessarily precise enough to obtain the desired information. In this work, different strategies are suggested to improve the versatility of BEoL robustness evaluation approaches.

**Fig. 1:**
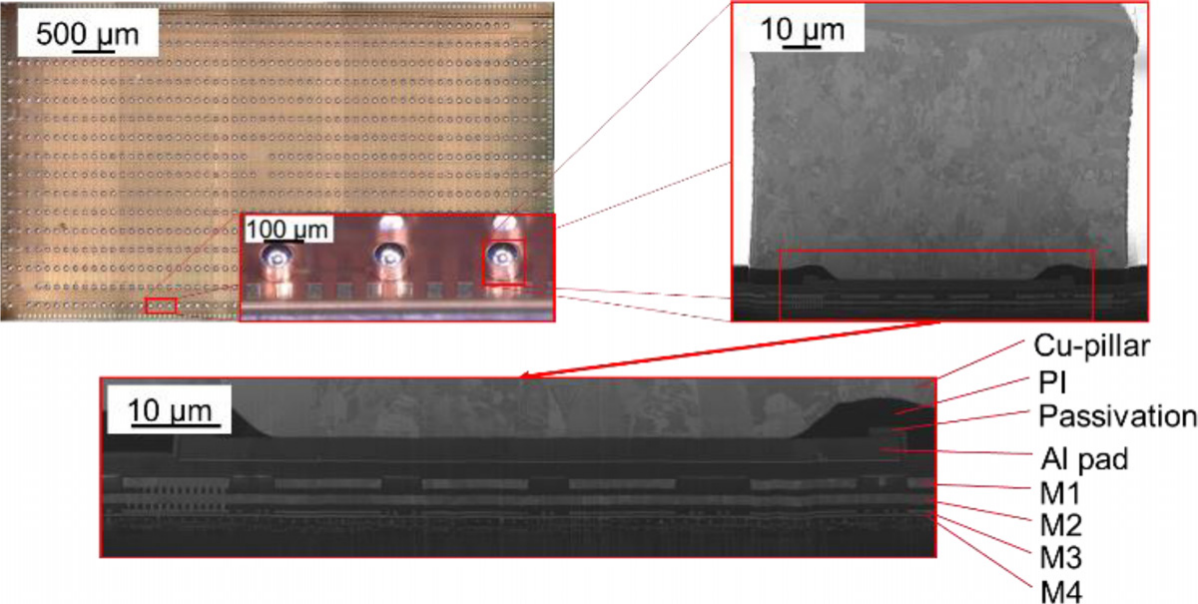
The basic TV investigated with the introduced methods [5].

Since the development objective of the presented methods was the thorough investigation of a specific Microchip, the measurement setups were designed for this specific TV (test vehicle). However, the approaches introduced in this work are not limited to this specific sample system but are more broadly applicable. Nonetheless, this specific sample system shall be briefly introduced in the following as it was utilized as TV for the described methods. The sample is a microchip with a length of 10 mm, a width of 5 mm and a silicon substrate thickness of 700 µm. It is bumped with 916 identical round Cu-pillars with a diameter of 95 µm and a height of 65 µm. Every Cu-pillar is capped with 35 µm of SnAg solder which results in a total height of 100 µm. The pitch between the Cu pillars is 150 µm in one and 350 µm in the other direction. An overview of the sample as well as the image of a FIB cut through the Cu-pillar and the subjacent BEoL stack is provided in Fig. 1.

**Fig. 2:**
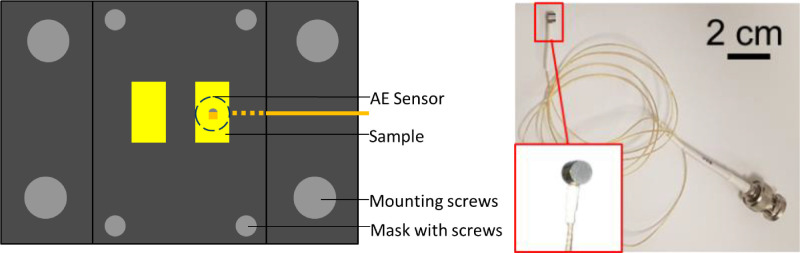
Schematic top view of the sensor/sample holder as well as the AE sensor [6].

To detect the occurrence of damage, AE (acoustic emission) signals are constantly monitored during the mechanical loading experiments. The advantage of AE is that it can provide additional information on damages occurring in the sample. The high sensitivity compared to the mechanical measurement system enables a more precise identification of occurring minor damages. To implement AE monitoring during a mechanical loading experiment it was required to design a combined sensor/sample holder to enable the establishment of a defined contact between AE sensor and sample and contain it during an experiment. The sensor/sample holder is schematically depicted in top view in Fig. 2.

**Fig. 3:**
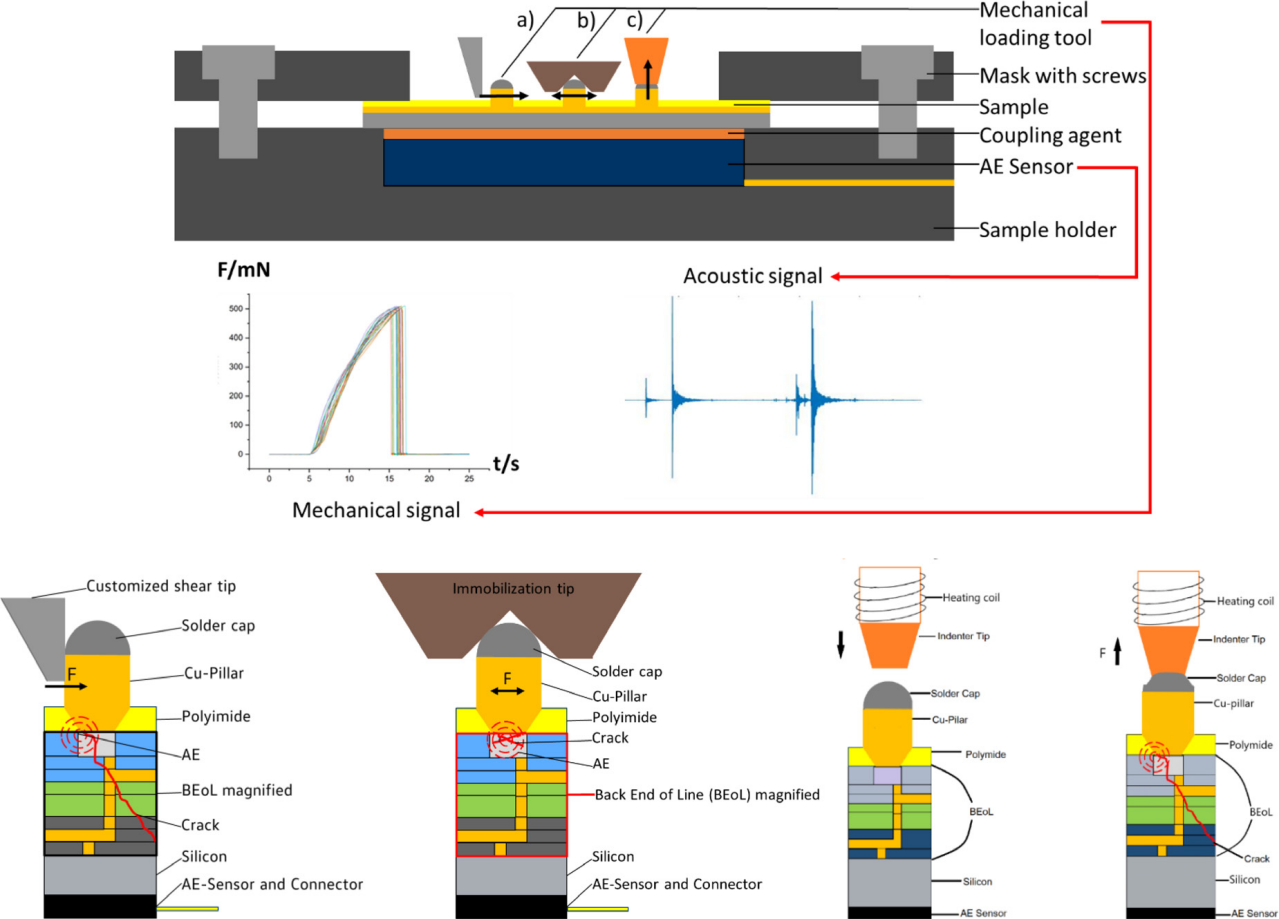
Depiction of the sensor/sample holder in side-view and the fundamental measurement concept for the three mechanical loading methods (a) Cu-pillar regular shear, (b) mechanical Cu-pillar immobilization, and (c) individual Cu-pillar soldering.

The design of the sensor/sample holder depicted in Fig. 2 is adapted to the Bruker/Hystitron TI950 measurement system which was utilized for most of the mechanical loading experiments. The holder is directly screwed to the base plate of the system which is sufficient to hold it in place during the experiment. A metal mask with two sample windows is utilized to immobilize the TV during the experiment. A schematic cross section of the sensor/sample holder along with the basic measurement concept of the three experimental approaches presented in this work, namely the regular shear, the mechanical immobilization and the soldering approach, are schematically depicted in Fig. 3.

The schematic lateral view of the sensor/sample holder depicted in Fig. 3 provides a better insight regarding the experimental setup. Firstly, the AE sensor is placed in a cavity in the holder of exactly the size of the sensor. Then, a coupling agent is applied to ensure the transmission of acoustic signals from the sample to the sensor. In this work high vacuum grease has been used for his purpose. The sample can then be placed on the sensor and immobilized with the mask. The stickiness of the high vacuum grease facilitated this process since it holds the sample in place. The intermetallic dielectric material as well as the silicon substrate itself are acoustically conductive, therefore the sensor can be attached to the back side of the sample. After screwing the holder into the indenter base plate or to another mechanical loading unit and connecting the AE sensor to the measurement system, experiments can be initiated. The basic measurement setup and strategy for the three mechanical loading approaches is essentially the same. The sensor utilized for the AE measurements is a Mistras PICO which can be applied to this measurement environment due to its small geometric features of only 5 mm diameter and 4 mm height. This sensor is sensitive for a frequency spectrum of several 100 kHz up to over 1 MHz and has a resonance frequency of 550 kHz. A measured signal is pre-amplified with a Mistras 2/4/6 Preamplifier at a rate of 60 dB. The amplified signal is then fed into an HBM transient recorder and measured with the custom Software “Perception”. During the application and simultaneous measurement of mechanical load, the AE events are monitored but only measured as soon as the amplitude surpasses a certain threshold. To obtain a high signal resolution of 100 MSamples/s, data is not continuously acquired but only in case an AE event occurs due to memory limitations. In order not to lose any parts of the signal, up to a couple of seconds of it can be saved in a loop. They are continuously overwritten until the threshold for a measurement is crossed. The threshold is typically set to 50 mV whereas a Cu-pillar delamination event triggers signals of more than 5 V. However, especially the small signals should be recorded since they indicate minor initial damage. These events can provide information regarding the mechanically most fragile features or areas of a given sample. After the acquisition process, the data was processed utilizing MATLAB or Python scripts. The analysis of the measured AE signals can provide further information on the damage occurrence and with enough data, it should be possible to identify specific acoustic footprints. In this best case, this would enable the identification of specific damage modes only based on the acoustic analysis which would be a significant simplification of the damage mode identification process. This approach has been explored to a certain extend and results are provided in [7] and [8]. However, the architecture of a BEoL stack and the related damage procedures are highly complex, therefore this approach was not suitable at this stage to enable a distinct identification of the occurrence of damage of a specific type but only provide estimations.

The geometric properties of the TV required a miniaturization of the mechanical loading tools since the mechanical BEoL evaluation experiments should be conducted on single Cu-pillars. Initially it was attempted to conduct BEoL robustness experiments utilizing common indenter tips such as Berkovich and cube corner. However, due to the inclines of these tips, the induced mechanical loading conditions are difficult to control. Therefore, an indenter tip for the regular shear approach was manufactured from diamond with a 50 µm x 50 µm base to be deployed in the small gaps between the Cu-pillars. Another, very sharp tip with a base of only 300 nm was also manufactured but it proved to be too fragile to be in use for long. For the given sample, the 50 µm x 50 µm indenter tip was completely sufficient, for smaller Cu-pillars or µPillars or for shorter pitches, an indenter tip with a smaller base area is required. The most important feature of this tip is that at least one edge does not contain and inclines. The shear face of the tip should be parallel to the Cu-pillar to not induce and compressive stress into the BEoL stack as it is for example the case with the utilization of a Berkovich tip. This approach is not exactly a novelty, as mentioned before it is commonly applied in one way or another to evaluate the robustness of solder connections or subjacent structures. However, to obtain additional information regarding the occurring damage processes it is combined with the AE measurement approach. Mechanically, this loading method offers only two customizable parameters, shear velocity and shear height. The parametrization can be adapted utilizing the Bruker/Hysitron control software. All experiments were conducted displacement-controlled which means that the shear velocity was set over a defined shear distance and shear time. It could however not be determined that the shear velocity had a major impact on the occurrence of a specific damage mode. The shear height on the other hand has a significant influence which has been explored also regarding the induced AE signals in [8,9]. Considering the application conditions of an FC (flip chip) attached microchip, it is apparent that it is not possible to emulate these conditions utilizing the regular shear method. In an application, the movement of a Cu-pillar is confined by the solder connection between Cu-pillar and substrate (and additionally by underfill).

**Fig. 4:**
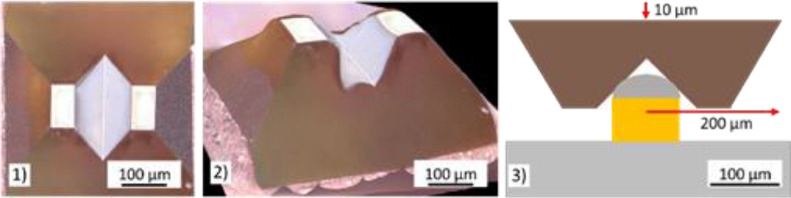
Indenter tip geometry and basic experimental approach of the Cu-pillar immobilization approach [5].

Considering this limitation led to the development of the mechanical immobilization approach. A new indenter tip with a customized design was manufactured to enable the immobilization of individual Cu-pillars in order to emulate the application conditions more closely. The geometry of this indenter tip as well as the basic experimental approach are provided in Fig. 4.

To establish an efficient immobilization, it is required to bring the inclined edges of the indenter cavity in contact with the Cu-pillar. To do so, an indent of 10 µm can be conducted which plastically deforms the comparably soft solder cap and mechanically connects the Cu-pillar to the indenter tip. A value between 8 and 12 µm should be selected for this indent. Since the Cu-pillar is much stiffer than the solder cap it will induce significant tensile force into the BEoL stack in case of an indent with high displacement. If the displacement is too small, the Cu-pillar is not adequately immobilized by the indenter tip. During a shear experiment only one inclined edge of the tip would be in contact with the Cu-pillar which would allow the latter to tilt. This would essentially lead to the same mechanical loading scenario as for the utilization of a cube corner tip. Due to its symmetrical shape, this indenter tip also enables cyclical loading experiments along one axis. The results of Cu-pillar shear-off and cyclical loading experiments conducted deploying this approach are presented in [10] and [11].

Even though the immobilization approach emulates the initial mechanical conditions of an FC soldered Cu-pillar closer as the regular shear approach, this technique exhibits several shortcomings. Due to the inclines of the cavity of the indenter tip, it is unavoidable that a compressive force is induced into the BEoL stack during a lateral loading experiment. Even though this apparently doesn't significantly damage the BEoL stack (at least it hasn't been identified in [10] and [11]) it is impossible with this approach to induce tensile stress. Tensile stress however does indeed occur under assembly and application conditions due to a mismatch of the coefficient of thermal expansion between the chip and the substrate. The damage mode also differs from the ones induced by other mechanical loading approaches. For instance, the cratering mode does not occur in case of the mechanical Cu-pillar immobilization approach as has been shown in [10] and [11], presumably because it is triggered by tensile stress in the BEoL stack. And especially this damage mode has been identified in experimental FC applications. Therefore, the method is considered not necessarily suitable to emulate experimental conditions which induce damages as they occur under assembly or application conditions. These considerations led to the subsequent development of the Cu-pillar soldering method. This approach should emulate the assembly process and therefore establish exactly the same mechanical conditions as in a FC application. With the soldering method it should be possible to cover almost all mechanical loading conditions of the immobilization approach. However, the immobilization approach would still be an adequate procedure for specific experimental conditions, e.g. in which the induction of heat stress is not desired or in which the BEoL robustness exceed the robustness of the solder connection.

**Fig. 5:**
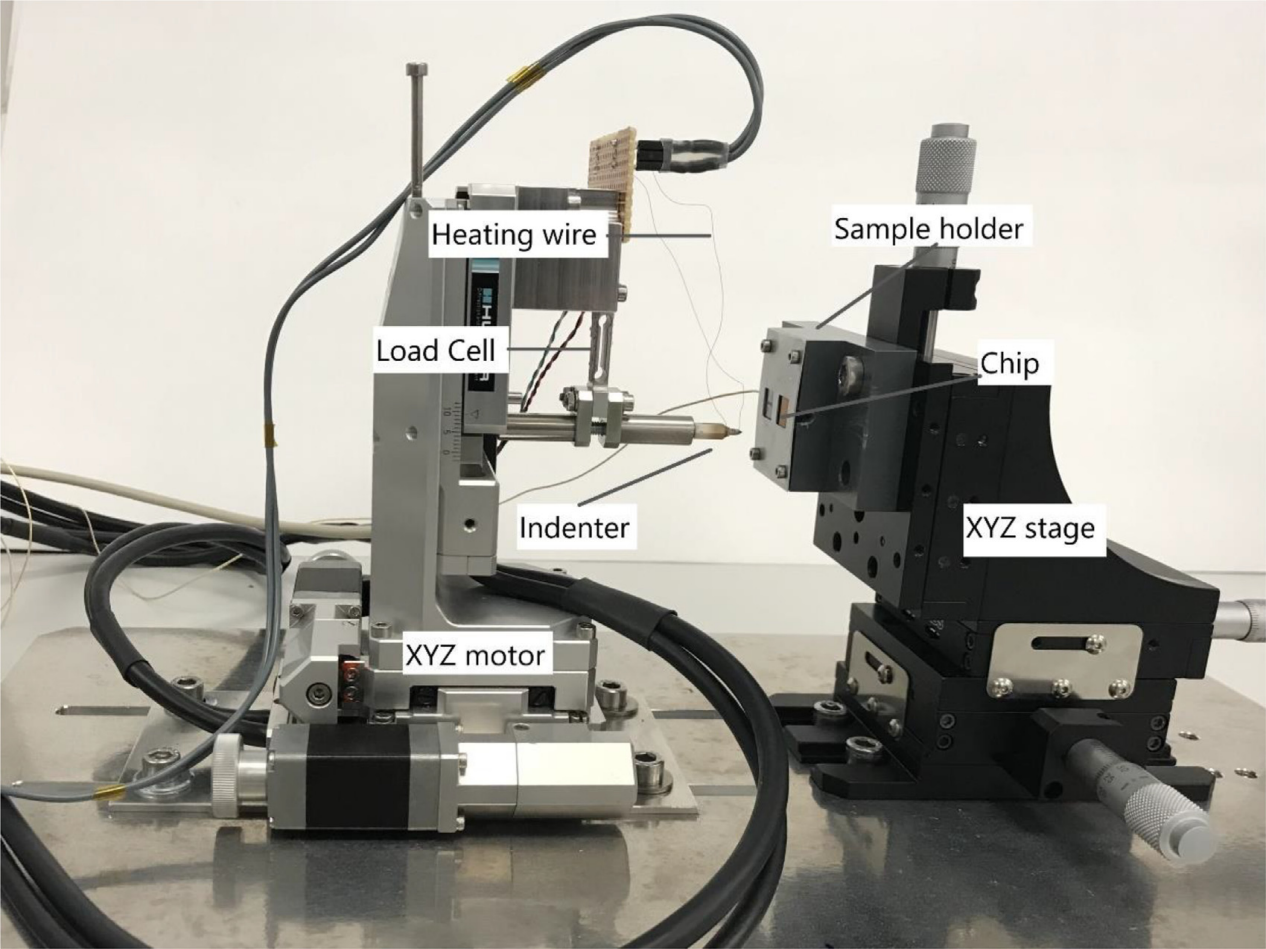
Developed soldering setup consisting of two XYZ stages each supporting the indenter and the chip respectively (USB camera not in image).

The most advanced and most promising development presented in this work is therefore the soldering approach. For the realization of this method, a new setup needed to be designed. The design is a mockup of a conventional triboindenter but based on horizontal movement instead of vertical movement. The assembled setup is depicted in Fig. 5.

As shown in Fig. 5, the setup consists of two main bodies, one part (left) for the indenter tip as well as the load measuring device and the other part (right) for the chip holder and the AE sensor. An identical sensor/sample holder was deployed for this approach as for the mechanical loading experiments conducted in the triboindenter system. To enable a maximum degree of freedom of motion, both parts are built on a XYZ-stage. For the motorized stage on the left side, a Huber Series 5102 with an AXO controller unit including a WIZ125SR gateway module was deployed. The load cell is positioned vertically hanging from an immobilized body at one end and connected perpendicularly to the indenter tip, which can then approach the sample horizontally. For this purpose, a Micro Load Cell CZL639HD from Phidgets Inc. was utilized. It was calibrated with the Bruker/Hysitron TI 950 equipped with an Omniprobe 3D system and is presented in Fig. 7.

On the opposite part of the indenter system, the chip is held in vertical position, immobilized in the sample holder which exposes the chip surface whilst encapsulating the AE sensor acoustically connected to the silicon substrate side of the chip. The soldering process and the tensile test experiments are visualized through a Dino-Lite USB camera which can provide live footage about the progress. On the backside of the indenter tip connection, a counterweight was implemented to prevent the gravitational force from influencing the measurement. The occurring gravitational force could have been subtracted out by calibration, but it was considered that it might tilt the indenter tip slightly. This incline could have an influence on quality of the solder connection and influence the applied tensile loading conditions therefore the tip had to be leveled (Fig. 8).

**Fig. 6:**
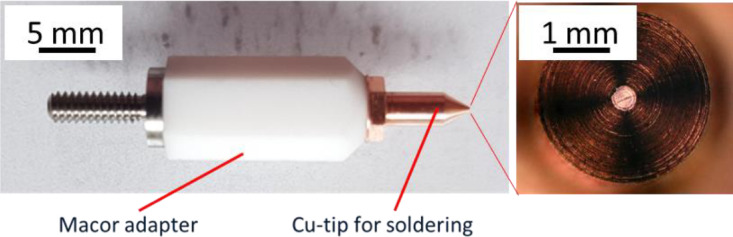
Custom-made soldering system with Cu tip (detail on the right) for soldering and Macor adapter for electrical and thermal insulation.

The objective of the Cu-pillar soldering approach is the emulation of firstly the assembly and secondly the application conditions of a specific microchip. By emulating the load condition on an isolated single Cu-pillar, a more precise evaluation of inflicted BEoL damage can be achieved. In this regard, a customized indenter tip was manufactured which can serve as a soldering bolt and as a landing pad for a solder connection at the same time. The indenter tip was designed also to be adaptable to a conventional triboindenter. The indenter system consists of two parts: Macor adapter and indenter tip. It is depicted in Fig. 6.

**Fig. 7:**
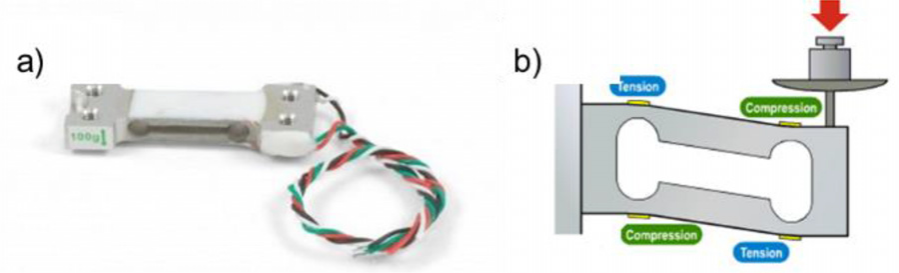
The Phidgets Inc. Micro Load Cell and functioning principle.

**Fig. 8:**
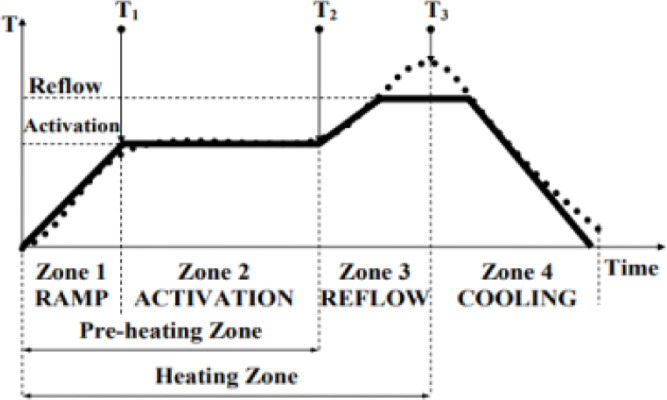
Typical temperature-time relation for reflow soldering [13].

The indenter tip has a cone shape with a tip diameter of 200 µm which serves as the landing area for the solder connection. The indenter shaft measures 2.5 mm in diameter and 5 mm in length. Its main purpose is to accommodate the heating wire coiled around it. The indenter tip and the shaft are made from one piece of copper to provide a high thermal conduction and at the same time ensure solderability. For the successful establishment of a solder connection between a Cu-pillar and the indenter tip, the latter needs to achieve reflow temperature which is 220 °C in case of the solder alloy Sn97Ag3 which is the one used in this TV [12]. In this regard, a Kanthal heating wire of 0.2 mm diameter connected directly to an electric power source is utilized. To provide electrical insulation for the indenter tip, 2–3 layers of Teflon tape are winded up in between the tip and the heating wire. The Teflon insulating tape needs to be changed when required which is approximately after 15 experiments. Due to the heat application, the tape slowly deteriorates and cannot provide electrical insulation anymore eventually. In this case the electrical current doesn't flow through the heating wire anymore but directly through the indenter tip. Therefore, the soldering bolt function is lost, and the electric current can even damage the sample system if in contact. For thermal and electrical insulation from the rest of the tool, a Macor adapter is installed. It doesn't only protect the connected systems from electrical or thermal damage, it also enables a better control over the soldering temperature at the indenter tip. Without the adapter, heat can dissipate through the copper tip and the metal thread into the periphery which in the developed approach is also manufactured from metal. This can lead to the situation that the soldering temperature is not reached at the copper tip even though it is insulated properly through the Teflon foil and the heating wire is brought to the required temperature.

In the following, the application procedure of the soldering approach is laid out. The heating profile during the soldering procedure can be controlled with the electrical current of the heating wire. An industrial scale chip packaging follows a specific heating profile that consists of five stages depicted in Fig. 2: Raising the heat to preheat temperature 3 °C/s, holding the temperature at preheat temperature for 60–120 s, raising the heat to reflow temperature 3 °C/s, reflow liquidation, and cooling to room temperature 3 °C/s [13].

For the developed setup introduced in this work, a more simplified version of the temperature profile is utilized initially but can be adapted if required. Another factor for a successful soldering connection is the wettability of the reflow solder on the substrate. To assist the wetting of the surface and prevent copper oxidation, additional flux is used to cover the copper indenter tip before the initiation of the soldering procedure. The flux reduces surface tension and can withhold the molten solder and components for the duration of the reflow procedure in an encapsuled environment [14].

Utilizing the assembled setup, parametrization studies of the soldering approach were conducted followed by tensile loading experiments. To emulate the flip chip assembly, process parameters such as initial contact force, heating rate, soldering time and cool down time must be optimized. The experiment workflow is as follows with the beforementioned components:

1. Preparation of the indenter tip by applying a thin layer of flux utilizing a brush.
2. Approaching an unsoldered single Cu-pillar with an initial force of 150 mN – 400 mN, monitoring the positioning with the USB camera.
3. Application of an electrical current of 0.7 A and voltage of 2.3 V to the heating coil to increase the indenter tip temperature up to the reflow level. After reaching reflow temperature hold for 10 – 20 s.
4. Removal of the current followed by 1 min of cooling down allowing a solder connection to form between indenter tip and Cu-pillar.
5. Application of tensile force to the Cu-pillar through the motorized XYZ stage with constant speed until a failure occurs.
6. Removal of the detached Cu-pillar from the indenter tip by reapplying heat and wiping with a Q-tip once the solder is molten.

From stage 3 to 5, AE signal measurements were conducted simultaneously, and acoustic signals were recorded.

**Fig. 9:**
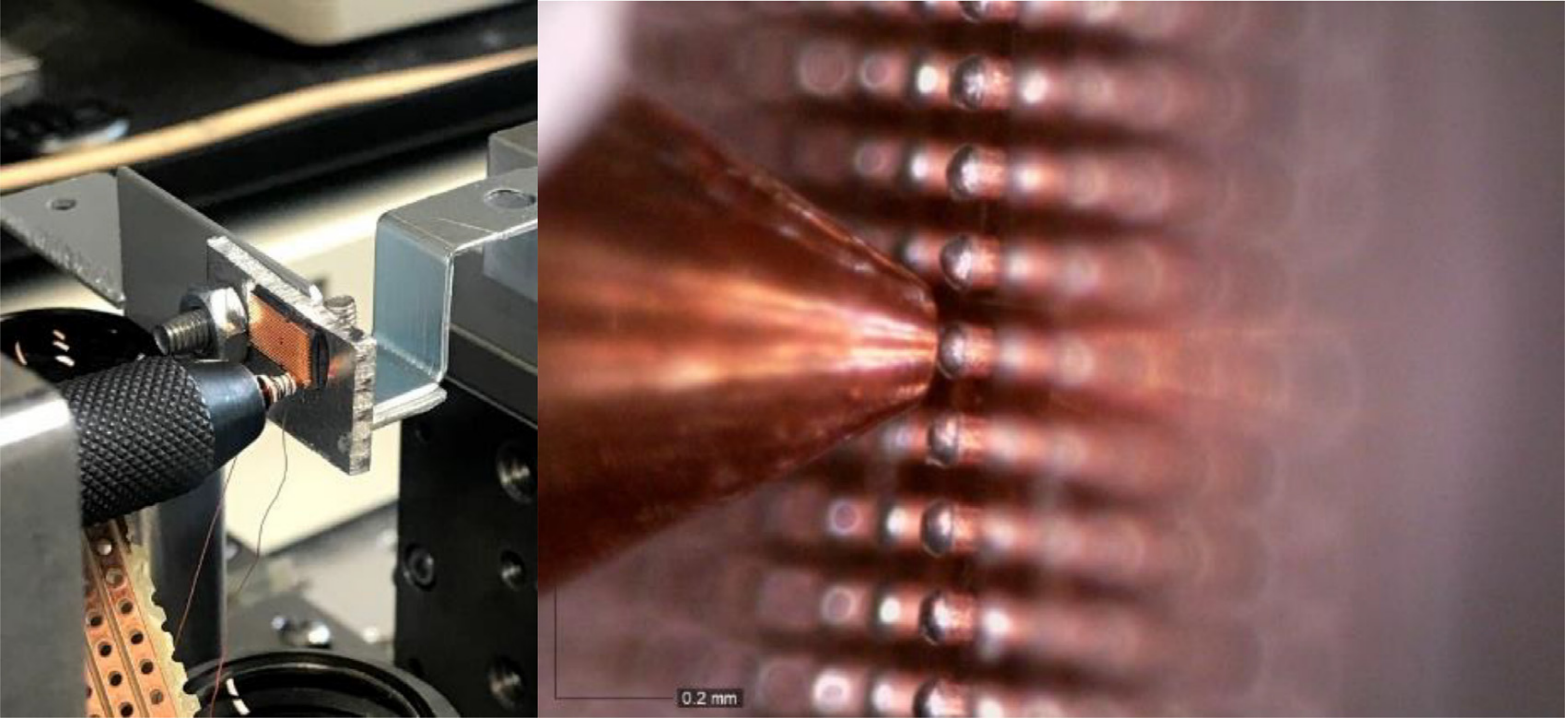
Screenshot of the USB camera video feed for the solder-tensile experiment.

It is important to apply a balanced initial force which can emulate the gravitational force during the packaging procedure without applying excess initial force. Another factor to consider for a successful tensile experiment is to remove the excess heat resulted from the soldering process. The chip's internal structure is mostly consisting of Cu and Si which are good heat conductors, therefore it is important to wait a sufficient amount of time for solder to form and for heat to dissipate completely from the chip. An overview of the soldering process and a detailed image derived from the live footage of the USB camera are provided in Fig. 9.

The integrity of the solder connection is defined by the width and quality of an IMC (intermetallic composition) layer forming between the copper indenter tip and the solder cap during reflow soldering. However, during multiple reflows, the IMC layer stays on the copper indenter tip surface which only grows further and decreases the solderability. As the thickness of the IMC grows, its brittle nature causes the mechanical strength of a new solder connection to deteriorate. A postmortem EDX analysis was conducted on the cross section of IMC/copper indenter tip once the soldering process becomes no longer possible due to the residue IMC accumulation. The IMC then has to be mechanically removed through a polishing process. This is the main drawback of the Cu-pillar soldering approach. All in all, the soldering method enables a high level of customizability regarding the induction of different mechanical loading conditions such as shear, tensile, mixed, or even torsion. Once the solder connection is established, every desired combination can be induced by specifically defined displacement. This enables the induction of customized mechanical loading scenarios on Cu-pillars and facilitates the emulation of specific desired mechanical loading conditions. Tensile, shear, even torsion load, and desired combinations can be induced to single solder joints, even with underfill in between. It enables the design of additional experimental approaches as well, for example the mechanical robustness testing in a specific temperature regime between room and soldering temperature to emulate high performance application conditions. Also, a variety of experiments for the assessment of the soldering/assembly parametrization are possible. The approach can be utilized to determine the influence and optimal values of soldering process parameters such as contact force, amount of flux applied, and temperature profile or also material characteristics such as soldering alloy composition or landing pad functionalization. The mechanical solder durability can also be determined through highly localized multiple reflow testing.

**Fig. 10:**
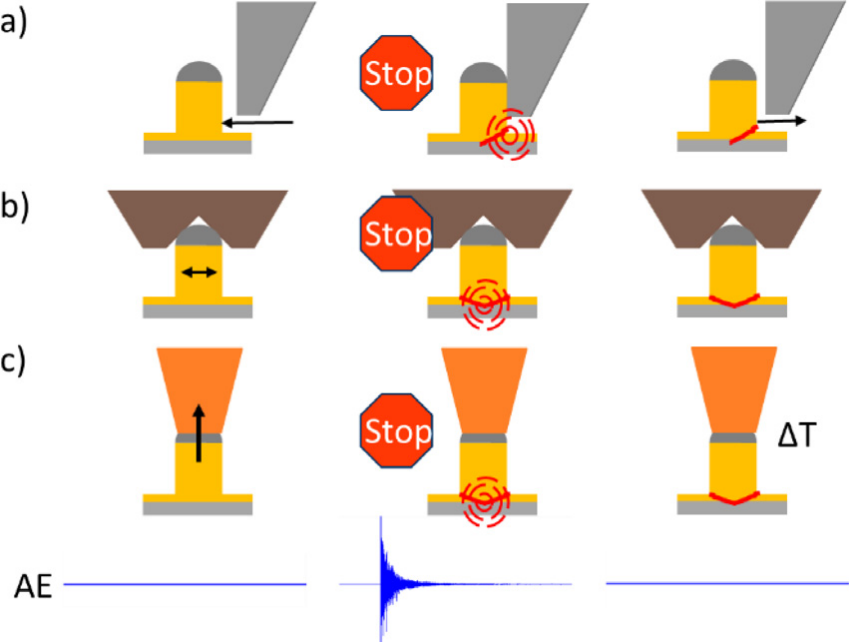
Sub-critical loading strategies of the three different mechanical loading methods utilizing AE monitoring as damage indicator.

The data generated with the presented methods enabled the determination of the failure mode under specific experimental conditions and the maximum forces under which the failures occur. However, the location of damage initiation and therefore the most damage prone component as well as the exact damage propagation sub-steps could not be identified. To do so, it was required to develop a sub-critical loading strategy. Due to the beforementioned high sensitivity of the AE measurement approach, acoustic signals are perfectly suitable to serve as a damage indicator. The amplitude of a respective AE signal enables also the differentiation between a sub-critical damage and a BEoL/Cu-pillar detachment event. Upton the detection of an AE signal during an experiment, the additional application of mechanical load is manually stopped in the triboindenter software. This could not be automated up to now due to the in incompatibility of the AE measurement system and the Hysitron/Bruker TI 950. The concept is schematically depicted for the three mechanical loading modes in Fig. 10.

**Fig. 11:**
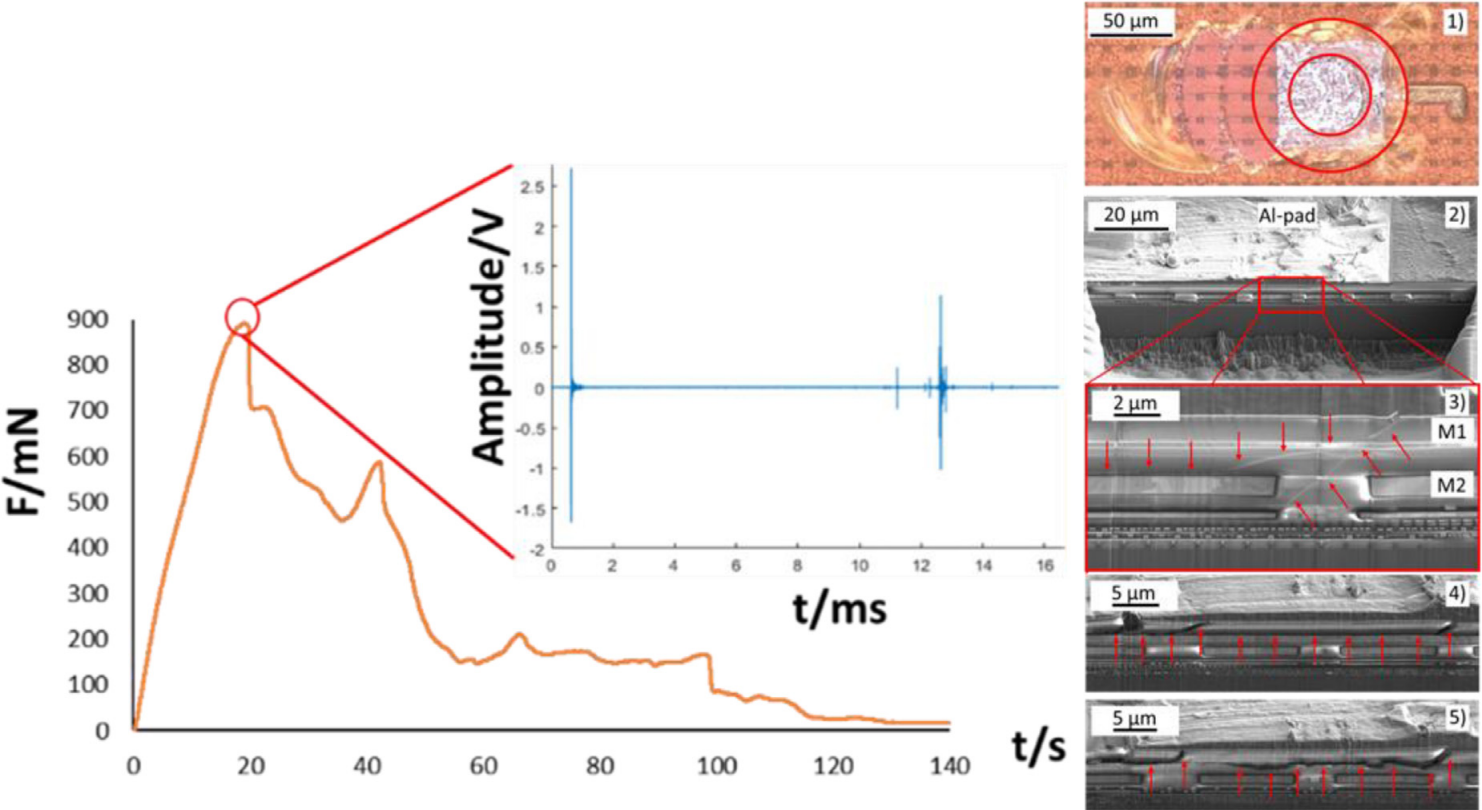
The shear force progression curve and corresponding AE signal of a Cu pillar shear-off event utilizing the mechanical confinement approach (left) as well as (1) optical microscopy overview and (2–5) FIB/SEM images of the shear-off experiment with damages indicated (red arrows) (right) [11].

To conduct sub-critical experiments, typically a low mechanical loading velocity (1 µm/s) was selected to enable the operator to react in a timely manner and abort the experiment. The velocity did not have an influence on the failure mode in case of the investigated sample. Applying this method enables the identification of the most damage prone area in a given TV. Applying this method enables the identification of the most damage prone area in a given TV. The physical process of damage and related AE signal occurrence inside the BEoL stack is always the same. However, due to the variation of mechanical loading conditions induced by the different methods, the damage location changes based on the induced stress fields and the location most prone for damage for a specific loading scenario. By deploying subsequent damage analysis techniques such as nXCT and SEM/FIB, the induced damages can be quantified. For sub-critical mechanical loading experiments utilizing the presented sample, initial damages were identified at the same location for all three methods which was the edge of the Aluminum contact pad. This has been presented in [6] for the regular shear approach and in [10] and [11] for the mechanical Cu-pillar immobilization and the soldering approach. The AE signals have always been utilized as a damage indicator and the experiments have been focused on the detection of initial AE signals and quantification of the related damage. This enabled the identification of the most damage prone areas and therefore the evaluation of damage mitigation strategies for a respective BEoL stack and loading condition. The mechanical and acoustic results as well as the resulting damage of a Cu-pillar detach experiment utilizing the mechanical confinement approach for lateral load induction are presented in Fig. 11.

**Fig. 12:**
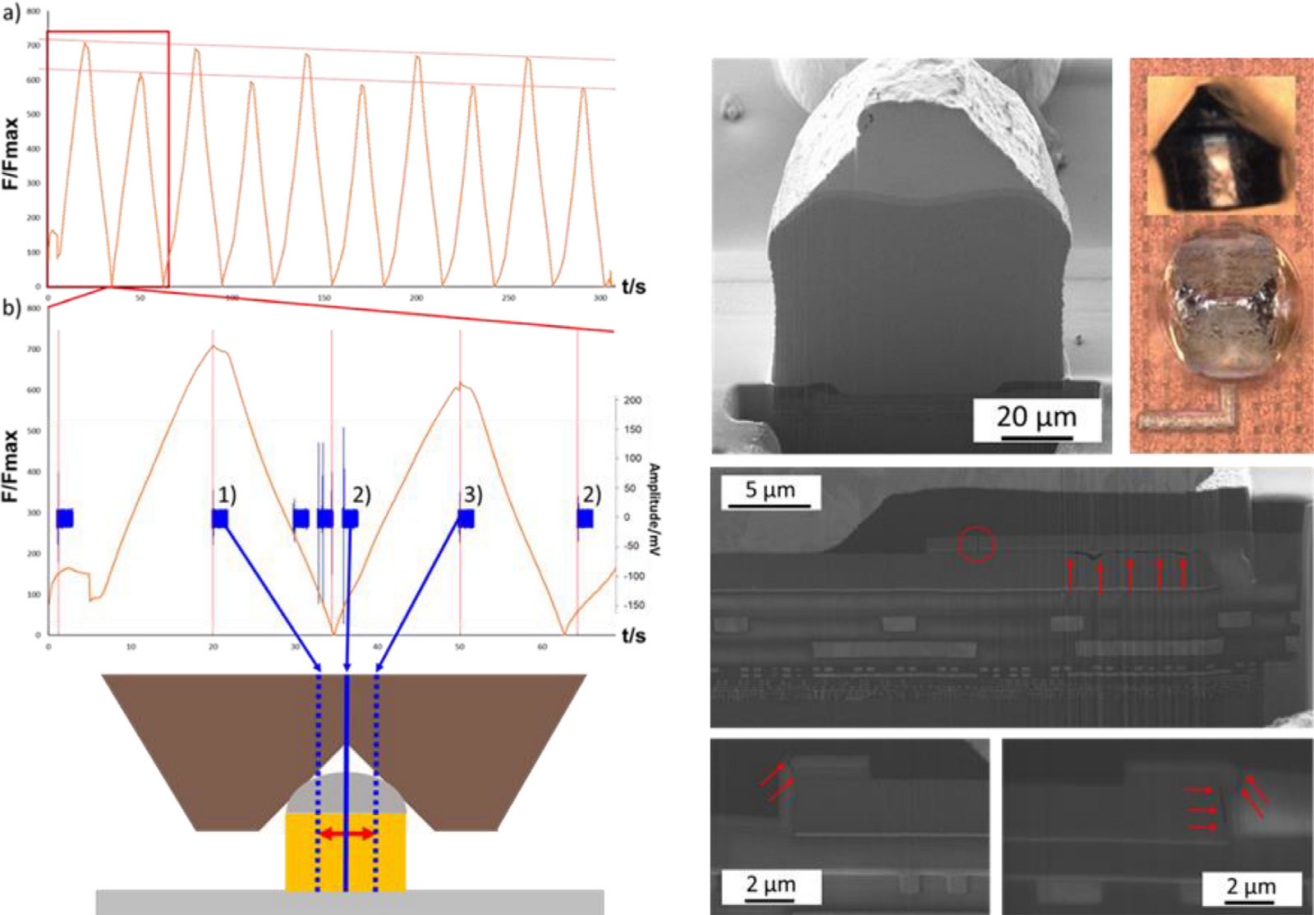
(a) The shear force progression curve overview of 5 loading cycles and (b) the detail view of one cycle with the corresponding AE signals and the related location of the shear tool (left) as well as images of optical microscopy measurements and the FIB/SEM analysis of a sub-critically loaded Cu-pillar with damages indicated (red arrows) [11].

As can be seen in Fig. 11 (left), the AE signal indicates the detach of the Cu-pillar which occurred at a lateral force of 890.5 mN. The lateral force doesn't drop to zero immediately afterwards since the Cu-pillar is confined through the indenter tip and scratches over the Polyimide passivation which covers the BEoL stack. This is clearly confirmed by the microscopy image. A subsequent SEM/FIB investigation revealed the damage induced to the BEoL stack. As can be seen in Fig. 11. (right), cracks propagate through the Aluminum contact pad and into the BEoL stack. The damage proliferates only through the low-k material and is deflected by the conductive Cu-grid. The damage origin could not be identified in this experiment. Therefore, a sub-critical cyclic loading experiment was conducted utilizing the mechanical confinement method.  The mechanical and acoustic results as well as the resulting damage of this experiment are presented in Fig. 12.

**Fig. 13:**
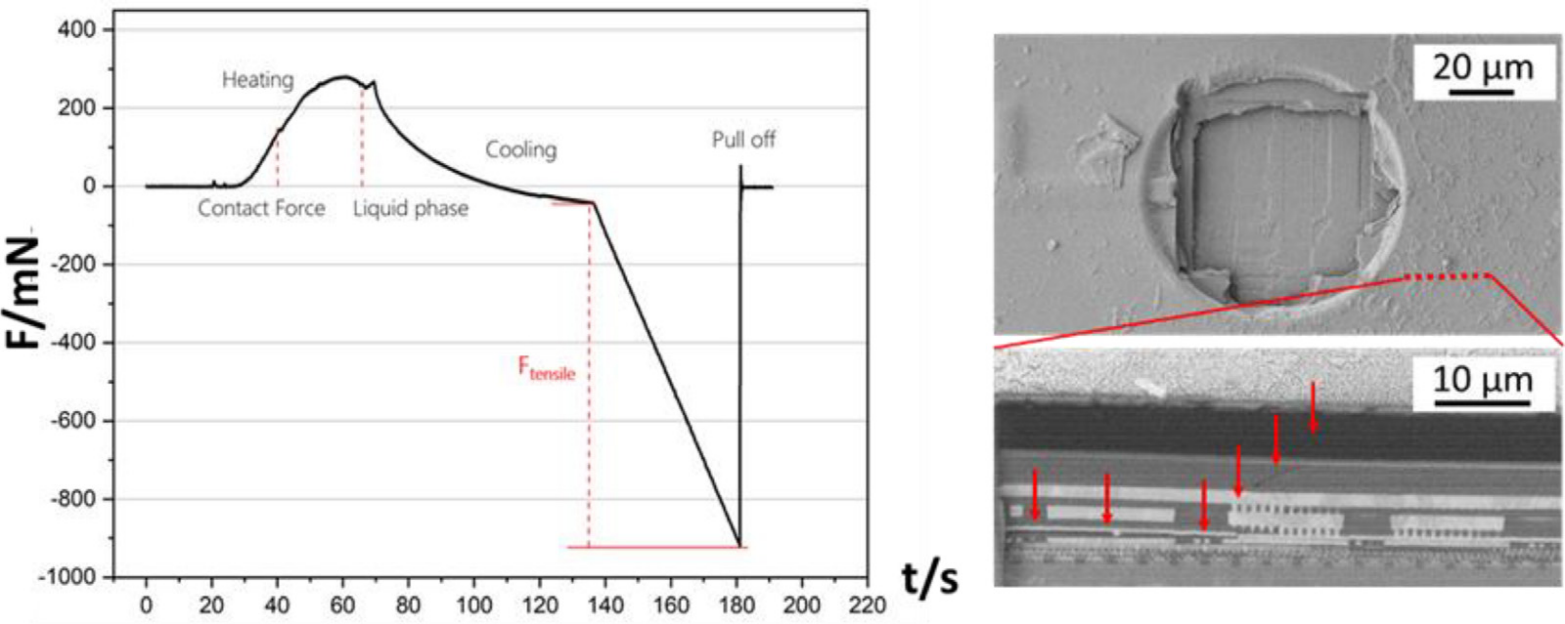
Force progression curve for solder-tensile experiment (left) as well as SEM overview image of the damage induced by the tensile experiment with FIB location indicated as well as FIB cross section with damages highlighted (right) [11].

The amplitudes of the AE signals measured during sub-critical loading are at least one magnitude smaller than the ones measured during the detach experiment. This indicates that only minor damage has been inflicted to the BEoL stack. The sub-critical loading experiment therefore enabled the identification of the damage origin for this loading condition as can be seen in the SEM/FIB damage analysis. The most damage prone area could be identified as the interface between the Aluminum contact pad and its passivation. Initial damages proliferate from there and lead to the detach of the Cu-pillar and crack propagation into the BEoL stack in case additional mechanical load is applied. Similar experiments have also been conducted utilizing the Cu-pillar soldering and tensile stress method. The force progression curve as well as the occurring damage of such an experiment are presented in Fig. 13.

**Fig. 14:**
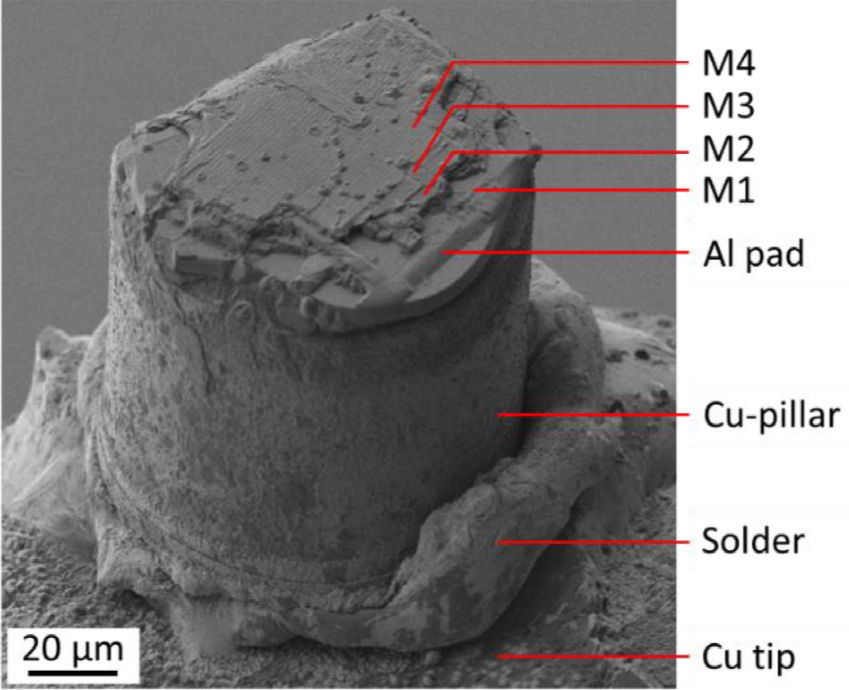
Detached Cu-pillar with residual BEoL stack after soldering and tensile loading experiment [11].

In case of a tensile experiment, the damage propagates much deeper into the BEoL stack which results in a cratering damage mode. The SEM/FIB analysis shows that cracks also propagate horizontally below the sample surface. The occurring damage is also strongly influenced by the geometrical characteristics of the Aluminum contact pad which conducts the tensile stress into the BEoL stack. The soldering method also enables the evaluation of the residual BEoL stack still attached to the Cu-pillar after a detach as depicted in Fig. 14.

**Fig. 15:**
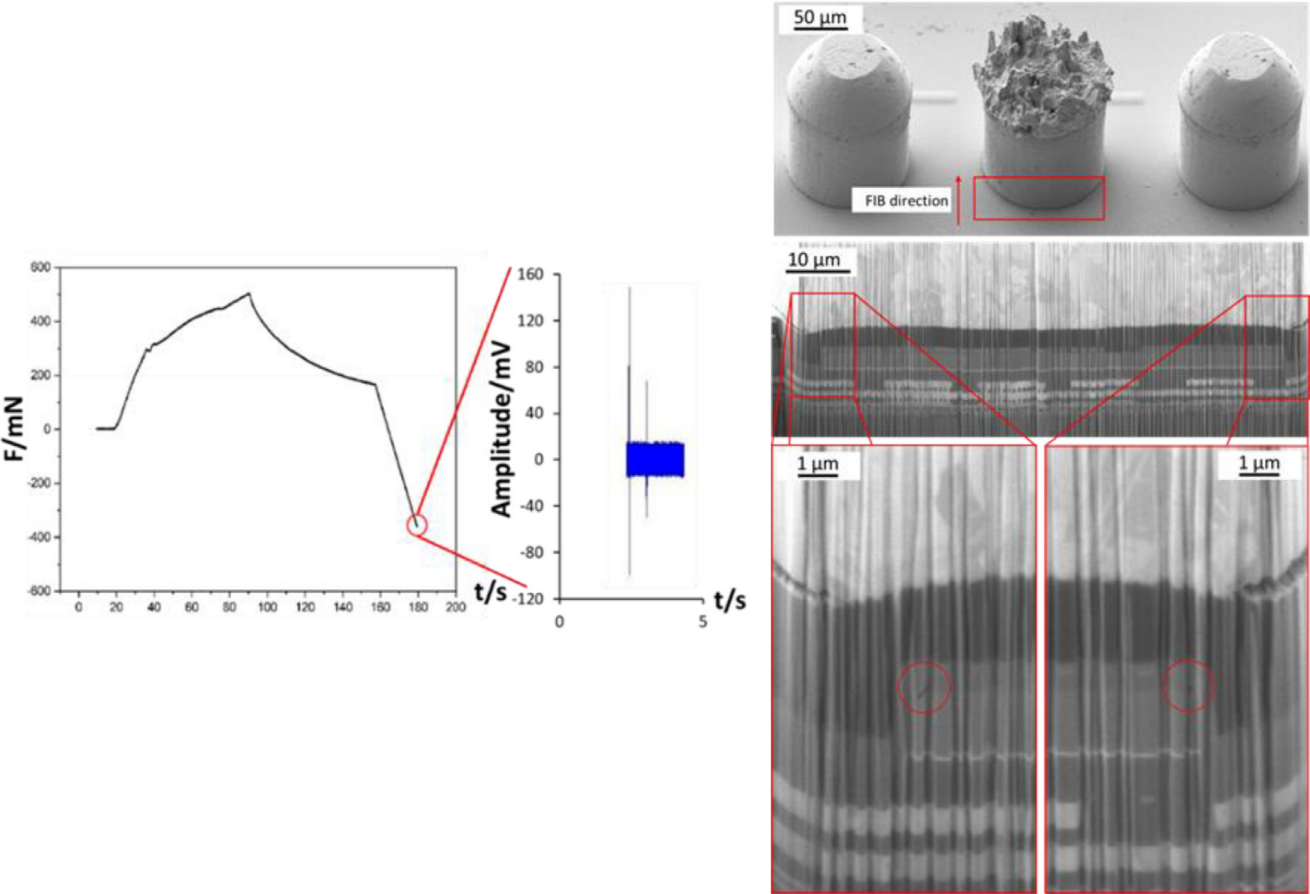
Force progression curve of the sub-critical tensile experiment and AE signal indicating damage (left) as well as FIB analysis of the sub-critical Cu-pillar tensile stress experiment (right) [11].

Conducting Cu-pillar detach experiments enables the evaluation of the final damage state but doesn't provide any information regarding the damage origin and proliferation. Therefore, it is required to conduct sub-critical loading experiments. The mechanical and acoustic result as well as the resulting damage of such an experiment are presented in Fig. 15.

Evaluating the results of the sub-critical soldering and tensile loading experiment enables the identification of the damage origin. For the tensile loading scenario, the most damage prone area is also the interface between Aluminum contact pad and its passivation. This result is similar to the one obtained with the mechanical confinement method, only that in this case especially the upper edges of the Aluminum contact pad seem to be affected.

**Fig. 16:**
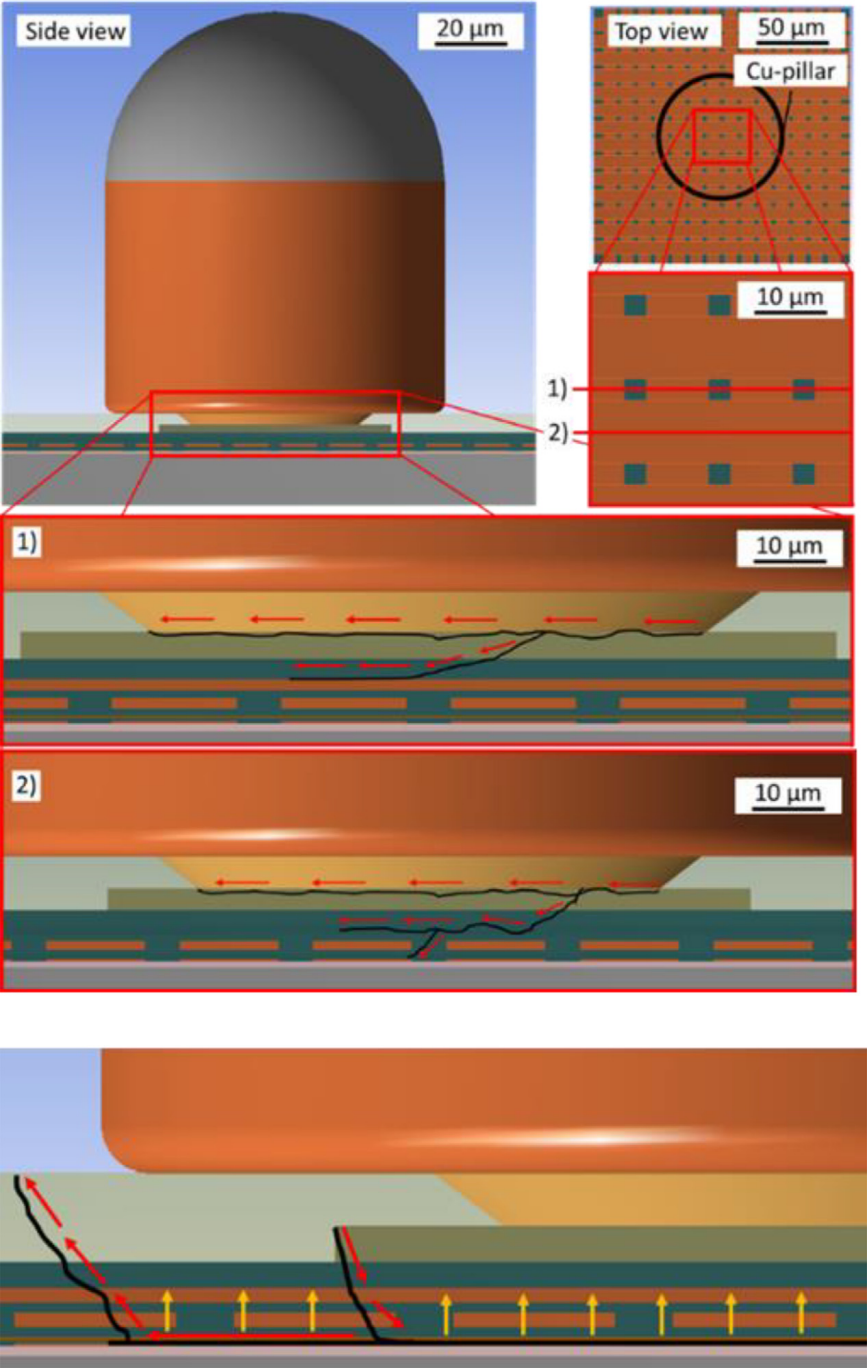
Damage propagation as determined in case of the mechanical immobilization approach (top) as well as for the soldering and tensile loading approach (bottom) [11].

Utilizing the sub-critical mechanical loading strategies enabled the identification of different damage processes occurring at the same time which could've not been done without the sub-critical investigation. Therefore, especially the sub-critical measurement approach has proven to be an adequate tool to support the quantification of damages occurring under micromechanical load. It enables the identification of the location of damage initiation in a given BEoL stack and therefore the determination of the most damage prone area. It also enables the characterization of the damage proliferation. These results support the development of damage models for a given BEoL stack and mechanical loading scenario. This has been carried out for the mechanical immobilization and the soldering approach as depicted in Fig. 16.

The damage models as presented in Fig. 16 can be helpful for the implementation of BEoL design optimization measures. Even though the experiments have been conducted utilizing a specific TV, their application is not limited to this specific sample or even sample type.

Comparing the different methods, it is concluded that the soldering approach, even though it is more complicated to deploy, is the most advanced and versatile to determine the mechanical robustness of a BEoL stack by mechanically loading superjacent connectors. Especially this approach bears great potential for further robustness investigations of BEoL stacks or other electronic components as well as solder connections themselves since it can be utilized to emulate a great variety of mechanical loading scenarios.

## CRediT authorship contribution statement

**Jendrik Silomon:** Conceptualization, Methodology, Writing – review & editing. **Dulguun Chimeg:** Methodology, Writing – review & editing. **André Clausner:** Supervision. **Ehrenfried Zschech:** Supervision.

## Declaration of Competing Interest

The authors declare that they have no known competing financial interests or personal relationships that could have appeared to influence the work reported in this paper.

## Data Availability

No data was used for the research described in the article.
